# Structural Properties of Janus Particles with Nano- and Mesoscale Anisotropy

**DOI:** 10.3390/nano10050989

**Published:** 2020-05-21

**Authors:** Eugen Mircea Anitas

**Affiliations:** 1 Joint Institute for Nuclear Research, Dubna 141980, Russia; anitas@theor.jinr.ru; 2 Horia Hulubei, National Institute of Physics and Nuclear Engineering, 077125 Bucharest-Magurele, Romania

**Keywords:** Janus particles, small-angle scattering, structural properties, Monte-Carlo simulations

## Abstract

Synthesis of anisotropic Janus particles (AnJPs) is crucial for understanding the fundamental principles behind non-equilibrium self-organization of cells, bacteria, or enzymes, and for the design of novel multicomponent carriers for guided self-assembly, drug delivery or molecular imaging. Their catalytic activity, as well as many other chemical and physical properties are intimately related to the nano- and mesoscale structure. An efficient and fast in situ monitoring of the structural changes involves non-destructive techniques which can probe macroscopic volumes of multicomponent systems, such as small-angle scattering (SAS). However, the interpretation of scattering data is often a difficult task since the existing models deal only with symmetric AnJPs, thus greatly restricting their applicability. Here, a general theoretical framework is developed, which describes scattering from a system containing randomly oriented and placed two-phase AnJPs with *arbitrarily* tunable geometric and chemical asymmetries embedded in a solution/matrix of different chemical composition. This approach allows an analytic description of the contrast matching point, and it is shown that the interplay between the scattering curves of the two phases gives rise to a rich scaling behavior which allows extracting structural information about each individual phase. To illustrate the above findings, analytic expression for the scattering curves of asymmetric AnJPs are derived, and the results are validated by Monte-Carlo simulations. The broad general features of the scattering curves are explained by using a simple scaling approach which allows gaining more physical insight into the scattering processes as well as for the interpretation of SAS intensity.

## 1. Introduction

Anisotropic Janus Particles (AnJPs) are nano/meso-scale particles composed of two or more regions with controllable material distribution and different geometric, physical, and chemical properties. The intrinsic property of each individual region generally give rise to improved properties of AnJPs as a whole. With the incorporation of AnJPs in materials in which they are allowed to assemble into a master blueprint, many exciting new phenomena crucial for the design of novel multicomponent carriers for guided self-assembly [1,2], drug delivery [3,4] or molecular imaging [5] are possible. Important applications are envisioned in anisotropic catalysis [6], tuning the microstructure of emulsions [7], and quasiquadrupole interface distortions [8].

Of particular interest is the synthesis of materials based on catalytically active AnJPs with two phases. In such particles, one region promotes chemical reactions while the other one is inert, and thus such materials are ideal candidates to study the behavior of complex systems with more than one type of catalytically active particles, which share reactants or products. This may shed light on the fundamental principles behind non-equilibrium self-organization of cells, bacteria, or enzymes [9]. Such information potentially allows much greater control over a material’s properties, enabling the design rather than the selection of materials for specific uses [10].

It is well known that an effective design of materials with predefined functions and properties is intimately related to a detailed knowledge of the structure of AnJPs, besides the chemical composition. For example, Janus nanofibers with tunable structure in terms of width, interfacial area, and volume of each side are promising for fibrous guided tissue regeneration membranes, since anti-inflammatory agents, adhesive components for cell attachment, and nutritional ingredients can be loaded in them [11]. In addition, anisotropic Janus Si nanopillars arrays with modified specific surface functionalization provide new opportunities to control the flow and motion of fluids in microfluidic channels [12], while gold nanorod Janus membranes exhibit efficient and high-sensitive surface-enhancement Raman scattering and tunable plasmon resonance [13].

For multicomponent colloidal particles such as those based on AnJPs, the correlations between physical and structural properties are usually addressed by using small-angle scattering (SAS) either with neutrons (SANS) [14,15] or with X-rays (SAXS) [16,17,18]. Basically, it provides information about the size and shape, which is further used to develop structural models that can provide insight into their function and properties. It is an efficient, non-destructive, and fast probing technique of the structural changes occurring in macroscopic volumes. When combined with contrast-variation, SAS enables visualization of each individual component in AnJPs [19,20], thus making it a powerful tool for probing the interactions between various regions of AnJPs, as well as between each individual region and the solvent. In the case of SANS, the contrast-variation relies on the interaction of neutrons with hydrogen and its isotope deuterium, or on the dependence of the neutron scattering length of these hydrogen isotopes on their nuclear polarization [21]. For SAXS, the contrast variation is achieved by exploiting the fact that, for X-rays whose energy is close to the absorption edge of an element, the atomic scattering factor of that element is reduced by few electrons from its value far from the edge [22].

Although the structural properties of globular Janus particles can be accurately characterized by SAS [20], the structure of AnJPs can be described only for symmetric structures, i.e., for rods/disks in which the distribution of matter is symmetric with respect to the middle radial plane [23,24]. For such systems, semi-analytical expressions for the form factors have been determined, and have been used to provide a qualitative description of their morphology, i.e., elongated or planar, as well as for the radius of gyration. However, many experimental techniques have been developed recently to manufacture AnJPs in which the distribution of matter is not restricted to a particular symmetry such as in superstructures consisting from Janus cylinders in an antisymmetric configuration [25], Janus composites nanorods containing block copolymers [26], Janus microfibers with magnetic and fluorescence functionalities [27], and antibacterial PCL/PVP-AgNP Janus nanofibers [28].

In this work, the structural properties of cylindrical AnJPs with controllable geometric and chemical asymmetries are studied in both reciprocal and real space. The AnJPs consist from two distinct regions with arbitrarily densities situated asymmetric with respect to the middle plane parallel to the bases of the cylinder. In this context, the asymmetric AnJPs is a generalization of the Janus cylinder studied by Kaya [23]. For reciprocal space investigations, the SAS intensities of AnJPs are calculated analytically. The obtained expressions are used to obtain the size and shape of each individual region, as well as for derivation of analytic expressions of the contrast matching points. Monte Carlo simulations were performed to validate the developed models and to study their properties in real space, that is for determination of the corresponding pair distance distribution functions (pddfs), radii of gyration, and the positions of centers of masses of the two regions and of AnJPs as a whole.

For analysis of the scattering curves a simple scaling approach is used which allows understanding of the general features of the scattered waves. This approach is based on comparing the various successions of power-law decays to the various length scales of the two regions of AnJPs. In particular, it is applied to explain the rich scaling behavior arising from $q^{0}\to q^{-2}\to q^{-4}$ transitions, where $q=\left(4\pi /\lambda \right)\sin\theta$ is the magnitude of the scattering wave vector, λ is the wavelength of the incident radiation, and 2θ is the scattering angle. The conceptual simplicity of this approach allows gaining more physical insight into the scattering processes as well as for the interpretation of SAS intensity. The results demonstrate that a large range of experimental situations can be addressed within the proposed approach.

The paper begins with presenting a general background on SAS together with a description of the main quantities used throughout the paper such as cylindrical form factor, pddf, and radius of gyration $R_{g}$. Then, the main concepts and methods, i.e., scattering from multiphase systems and the Monte-Carlo method, are described. Further, the asymmetric cylindrical AnJPs model, derivation of the corresponding scattering intensities, and calculation of pddfs and radii of gyration from Monte-Carlo simulations are presented and discussed. Section 4 summarizes the main findings and discusses the implications of the obtained results.

## 2. Scattering from Three-Phase Systems

Let us consider a macroscopic sample consisting from a large number of scattering objects, randomly oriented and whose positions are uncorrelated. Then, by neglecting multiple scattering, the differential cross-section of elastic scattering is [29] $d\sigma /d\Omega ={\left|A_{\mathrm{tot}}\left(q\right)\right|}^{2}$, where $A_{\mathrm{tot}}\left(\cdot \right)$ is the total scattering amplitude, given by:

(1) $$A_{\mathrm{tot}}\left(q\right)=\int_{V^{\mathrm{irr}}}\rho_{s}\left(r\right)e^{iq\cdot r}dr.$$

In the above expression, $V^{\mathrm{irr}}$ is the volume irradiated by the incident beam (neutrons, light, X-rays), and the scattering length density (SLD) $\rho_{s}$ is given by $\rho_{s}\left(r\right)=\sum_{j}b_{j}\delta \left(r-r_{j}\right)$. Here, $b_{j}$ are the scattering lengths, $\delta \left(\cdot \right)$ is the Dirac’s delta function, and $r_{j}$ are the spatial positions of the scatterers.

For two-phase systems, one considers that the sample consists from stiff homogeneous objects and SLD ρ, immersed in a solid medium of SLD $\rho_{0}$. A constant shift of the SLD in the sample plays a role only at small values of scattering wave vector $q≲2\pi /{\left(V^{\mathrm{irr}}\right)}^{1/3}$. However, they are beyond the instrument resolution, since a typical irradiated volume is $V^{\mathrm{irr}}≃1$ mm ${}^{3}$, and thus $q≲10^{-6}$ Å${}^{-1}$. Then, by subtracting the background density $\rho_{0}$, one can consider the sample as if the scattering objects of SLD $\Delta \rho =\rho -\rho_{0}$ were fixed in vacuum. The quantity Δρ is called the scattering contrast.

**Figure 1:**
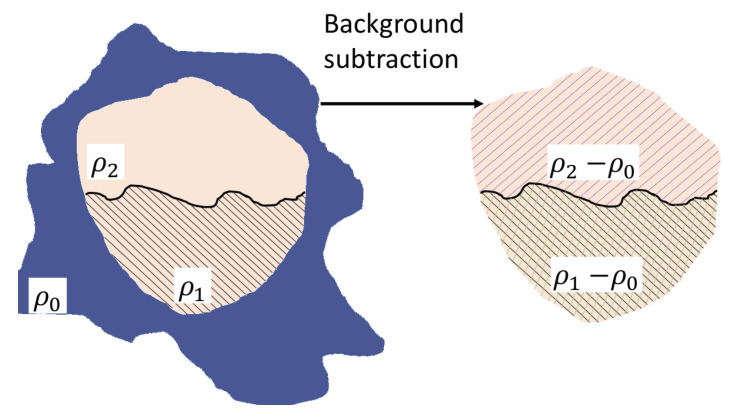
(Color online) Schematic representation of the background subtraction procedure. (Left) A three-phase system consisting from a particle of arbitrarily shape having two regions of different SLD $\rho_{1}$ and $\rho_{2}$, embedded in a matrix with SLD $\rho_{0}$. (Right) The corresponding two-phase system after subtracting the matrix background consists from the same regions but with SLD $\rho_{2}-\rho_{0}$ and $\rho_{1}-\rho_{0}$, respectively.

By considering AnJPs as scattering objects, one has to consider three-phase systems, due to the presence of two regions with different SLDs. To this aim, a simple approach initially developed for generalization of Stuhrmann method [30] is used here. The AnJPs used here, consist of a homogeneous region with SLD $\rho_{1}$ (Region 1) which contains another region with SLD $\rho_{2}$ (Region 2), and the whole particle is embedded in a matrix with SLD $\rho_{0}$. Figure 1 (left) shows a schematic representation of this model. Then, by performing the background subtraction as discussed above, the three-phase system is reduced to a two-phase one with contrasts $\Delta \rho_{1}=\rho_{1}-\rho_{0}$ and $\Delta \rho_{2}=\rho_{2}-\rho_{0}$ (Figure 1 right).

Therefore, the SAS intensity can be written as [29]:

(2) $$I\left(q\right)=n\langle {\left|A\left(q\right)\right|}^{2}\rangle ,$$

where A(q) is the scattering amplitude, and *n* is the concentration of AnJPs. Here, the brackets $\langle \cdots \rangle$ denote the mean value of the ensemble averaging over all possible orientations. Since the probability of each orientation is considered to be the same, the mean value is obtained by averaging over all directions of the scattering vector according to:

(3) $$\langle f\left(q_{x},q_{y},q_{z}\right)\rangle \equiv \frac{1}{4\pi }\int_{0}^{\pi }d\theta \sin\theta \int_{0}^{2\pi }d\phi f\left(q,\theta ,\phi \right),$$

where $q_{x}=q\cos\phi \sin\theta$, $q_{y}=q\sin\phi \sin\theta$, and $q_{z}=q\cos\theta$ are the components of the scattering vector in spherical coordinates.

It is well known that the normalized scattering amplitudes (form factors) can be written as $F\left(q\right)=V^{-1}\int_{V}e^{-iq\cdot r}dr$, where F(0)=1, and *V* is the volume of AnJP. Then, for the two-phase system shown in Figure 1, the scattering amplitude A(q) can be written in terms of the form factors $F_{1}\left(q\right)$ (corresponding to the overall particle, denoted Region 1) and of $F_{2}\left(q\right)$ (corresponding to the region with SLD $\rho_{2}-\rho_{0}$, denoted Region 2) [30]. Thus, when the form factor $F_{2}\left(q\right)$ is known, the scattering amplitude is $A\left(q\right)=A_{1}\left(q\right)+A_{2}\left(q\right)$. Here, $A_{1}\left(q\right)\equiv \left(\rho_{1}-\rho_{0}\right)\left[V_{1}F_{1}\left(q\right)-V_{2}F_{2}\left(q\right)\right]$ is the scattering amplitude of the region after subtracting Region 2 from Region 1, and $A_{2}\left(q\right)=\left(\rho_{2}-\rho_{0}\right)V_{2}F_{2}\left(q\right)$.

Then, the intensity (Equation (2)) can be written as:

(4) $$I\left(q\right)=n\langle |\left(\rho_{1}-\rho_{0}\right)V_{1}F_{1}\left(q\right)+\left(\rho_{2}-\rho_{1}\right)V_{2}F_{2}\left(q\right){|}^{2}\rangle .$$

By denoting

(5) $$\alpha \equiv {\left(1+\frac{\rho_{2}-\rho_{1}}{\rho_{1}-\rho_{0}}\frac{V_{2}}{V_{1}}\right)}^{-1},$$

Equation (4) becomes:

(6) $$I\left(q\right)=I\left(0\right)\langle |\alpha F_{1}\left(q\right)+\left(1-\alpha \right)F_{2}{\left(q\right)|}^{2}\rangle ,$$

where α is a dimensionless parameter which plays the role of a contrast parameter. Therefore, the scattering at zero angle can be written as:

(7) $$I\left(0\right)=nV_{1}^{2}{\left(\rho_{1}-\rho_{0}\right)}^{2}\alpha^{-2},$$

For the asymmetric AnJPs, Region 1 is a cylinder and Region 2 is also a cylinder, and thus the above approach is similar to the case of calculating the amplitudes of two cylinders and summing them up. However, its efficiency becomes clear when applied to AnJPs in which the distribution of the regions with different SLD takes a more complex form. For example, this approach could be used for studying the scattering properties of cylinder segments, where the scattering amplitude is more difficult to be calculated. In the remainder of the paper, we calculate analytically the form factor $F_{1}\left(\cdot \right)$, while the form factor $F_{2}\left(\cdot \right)$ is determined by the approach described above.

### 2.1. Scattering Amplitude from a Cylinder

**Figure 2:**
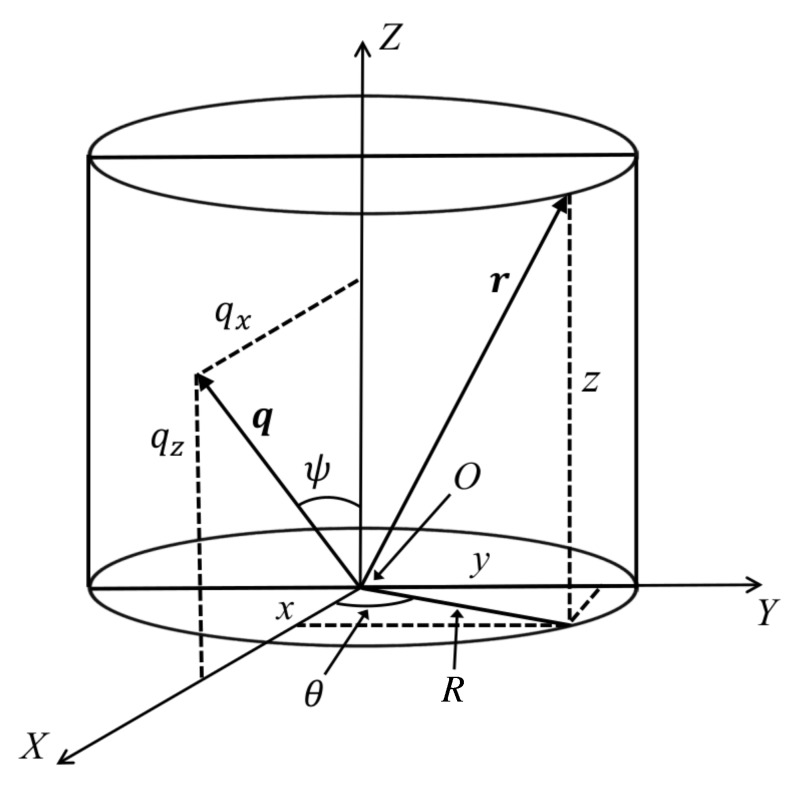
Representation of scattering and position vectors q (situated in XZ plane, i.e., $q_{y}=0$) and r in cylindrical coordinates, respectively. Here, $\left(x,y,z\right)$ and $\left(R,\theta ,z\right)$ are the rectangular and respectively cylindrical coordinates of the vector r in real space, where θ is the angle between the projection of vector r in the XY plane and the positive direction of X axis. The components of q are $\left(q_{x},q_{y},q_{z}\right)$ and $\left(q,\psi ,\zeta =q_{z}\right)$ in rectangular and cylindrical coordinates, respectively. ψ is the angle between the vector q and the positive direction of Z axis.

We start by considering a Cartesian system of coordinates, with $\left(x,y,z\right)$ and $\left(q_{x},q_{y},q_{z}\right)$ being the rectangular components of the position vector r and of the wave vector q, respectively. Thus, one can write that $r\equiv \left(x,y,z\right)$ and $q\equiv \left(q_{x},q_{y},q_{z}\right)$. A graphical representation of this situation is shown in Figure 2. Thus, for an arbitrarily function $\rho \left(r\right)$ the 3D Fourier transform can be written as:

(8) $$A\left(q\right)\equiv \int_{-\infty }^{\infty }\int_{-\infty }^{\infty }\int_{-\infty }^{\infty }\rho \left(r\right)e^{iq\cdot r}dr.$$

Here, dr=dxdydz denotes the volume element. Note that, in SAS, the function $\rho \left(r\right)$ represents a density of scattering volume for X-rays, or a SLD for neutrons, and thus $A\left(q\right)$ gives the corresponding scattering amplitude.

By positioning the scattering wave vector in the XZ plane (see Figure 2), its components become $\left(q\sin\psi ,0,q\cos\psi \right)$, and the scalar product is given by $q\cdot r=q\left(R\cos\theta \sqrt{1-\mu^{2}}+\mu z\right)$, where μ≡cosψ. Therefore, Equation (8) can be rewritten as:

(9) $$A\left(q\right)=\int_{0}^{\infty }\int_{0}^{2\pi }\int_{-\infty }^{\infty }\rho \left(R,\theta ,z\right)e^{iq\left(R\cos\theta \sqrt{1-\mu^{2}}+\mu z\right)}RdRd\theta dz.$$

### 2.2. Pair Distance Distribution Function and the Radius of Gyration

For calculating the radius of gyration, it is convenient to write the scattering intensity in terms of pddf as [29]:

(10) $$I\left(q\right)=4\pi \int_{0}^{D}p\left(r\right)\frac{\sin qr}{qr}dr,$$

where $p\left(r\right)=r^{2}\gamma \left(r\right)$, γ(r) represents the correlation function, and *D* is the maximum distance between two points inside the particle. Thus, pddf is related to the number of lines with lengths between *r* and r+dr joining distinct volume elements. For r=0 and r>D, p(r)=0, and therefore p(r) describes a histogram of distances but it does not contain any information about the orientations of these lines joining arbitrarily two points.

The sine term in the last equation can be approximated by a Taylor series expansion, and after some basic algebra, it can be easily shown that the radius of gyration can be expressed as:

(11) $$R_{g}^{2}=\frac{\int_{0}^{\infty }p\left(r\right)r^{2}dr}{2\int_{0}^{\infty }p\left(r\right)dr}.$$

In the case of a cylinder of radius *R* and height *H*, it can be shown that the radius of gyration is given by [29]:

(12) $$R_{g}^{2}=\frac{R^{2}}{2}+\frac{H^{2}}{12}.$$

### 2.3. Monte Carlo Simulations

Let us consider that *r* is the distance between two random points *t* and *u* of given coordinates. Then, the pddf is generated from the particle shape and composition, and the scattering intensity is calculated by performing a Fourier transform, according to Equation (10). Since the AnJPs are multiphase systems, we follow the procedure first described in [31], which consists in setting the number of random points $k_{1}$ and $k_{2}$ generated in Regions 1 and 2, respectively, and with contrast densities $\Delta \rho_{1}$ and $\Delta \rho_{2}$, to be proportional to the product $V_{1}\Delta \rho_{1}$, and $V_{2}\Delta \rho_{2}$, respectively.

Then, a weight $w_{i}$ of +1 is assigned to each coordinate inside AnJPs only if the contrast at this point is positive. Otherwise, a value of −1 is assigned if the contrast is negative. Finally, the distances between the points *t* and *u* are weighted by the product of the weights $w_{t}w_{u}$, followed by binning into frequencies as a function of *r*. This gives exactly the pddf p(r), which is used in Equation (10) to calculate the scattering intensity. As such, p(r) is proportional to the probability of finding two points at a distance *r* apart, and is normalized such that $\int_{0}^{D}p\left(r\right)dr=1$, where *D* is the maximum distance between any two random points in the AnJPs, as described above.

Thus, the property that, at r=0 and r>D, we have p(r)=0, is explained by the absence of any other volume element and because there are no distances bigger than *D* inside the AnJPs, respectively. Since there is no information about the orientation of the lines connecting the points within the particle, due to the spatial averaging, the scattering intensity is calculated by Equation (10), for arbitrarily values of the scattering wave vector *q*.

## 3. Asymmetric Anisotropic Janus Particles

### 3.1. Model

**Figure 3:**
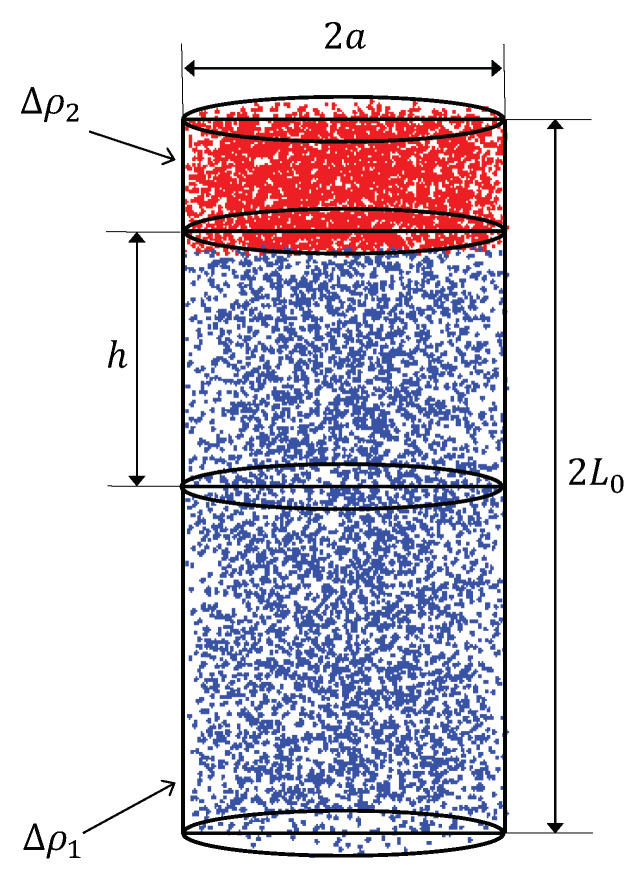
(Color online) The model of a axially AnJPs with two regions of different sizes and different SLD. Here, *a* is the cylinder radius, $2L_{0}$ its height, and *h* is the height above the middle-plane. Region 1, red points; Region 2, red and blue points.

The axially AnJPs consist from a cylinder of radius 2a and height $2L_{0}$. This is Region 1 (red and blue points in Figure 3) and thus has the volume $V_{1}=2\pi L_{0}a^{2}$. In this model, Region 2 described above is also a cylinder with the same radius, but height $L_{0}-h$ (red points in Figure 3). Here, *h* is the distance between the middle-plane parallel to the base of Region 1 and lower base of Region 2. It can be varied to arbitrarily value between 0 and $L_{0}$. The model is represented by using the random points generated using Monte-Carlo simulations. Thus, red points belong to Region 2, and blue ones to its complement. With these notation, the volume of Region 2 can be written as:

(13) $$V_{2}=\pi a^{2}\left(L_{0}-h\right).$$

### 3.2. Pair Distance Distribution from Monte Carlo Simulations

**Figure 4:**
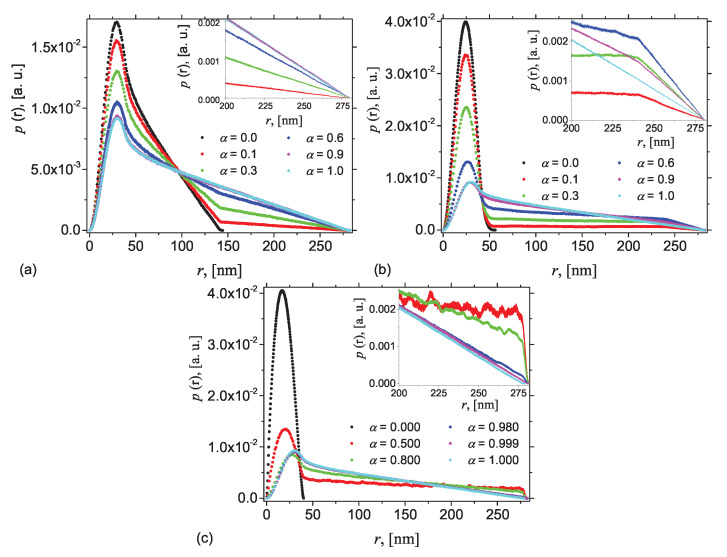
(Color online) Pddf p(r) from radially symmetric cylinders with diameter 2a=40 nm and height $2L_{0}=280$ nm calculated using the Monte-Carlo simulations, when Region 2 is found at various heights *h* above a middle-plane parallel to the bases: (**a**) h=0 nm; (**b**) h=100 nm; and (**c**) h=137 nm.

The corresponding pddf at fixed values of the height *h* and various values of the contrast parameter α are shown in Figure 4. The values of α≠0 have been obtained by considering that $\rho_{0}=0$ and $\rho_{2}=2$ in Equation (5), while $\rho_{1}$ is varied. For α=0 and α=1, the AnJPs are reduced to homogeneous cylinders, and the corresponding pddfs are calculated using the classical approach involving a random distribution of points in Region 2 and in Region 1, respectively.

The simplest case is presented in Figure 4a, which shows the pddfs at h=0, i.e., when Region 2 is half as compared with Region 1. At α=0, AnJPs reduces to Region 2 and the corresponding pddf has the well-known behavior where a peak, corresponding to the cross section, gives the largest number of distances that occurs, and is followed by a straight line up to $r=2L_{0}$ (black dots). The inflection point at r≃2a=40 nm gives a rough indication for the size of the cross section. However, as soon as α≠0, a second inflection point where the pddf increases its slope, occurs at $r=L_{0}+h=140$ nm. This is because the density of the complement of Region 2 is different from the density of Region 2 itself. As α is increased further, the differences between the slopes decrease, indicating the formation of a more homogeneous structure, in which the differences between the densities become less. The inset in Figure 4a shows that at large values of *r*, the differences between pddfs corresponding to α=0.9 and α=1 are almost indistinguishable. Finally, when α=1, the crossover point disappears, revealing the formation of a completely homogeneous structure. Note that the height of the maxima also decreases with increasing α, reflecting a decreasing of the maximum number of distances when increasing the contribution of Region 1. Thus, the differences between the slopes can be used to quantify the differences between the densities of the two regions in AnJPs.

**Figure A1:**
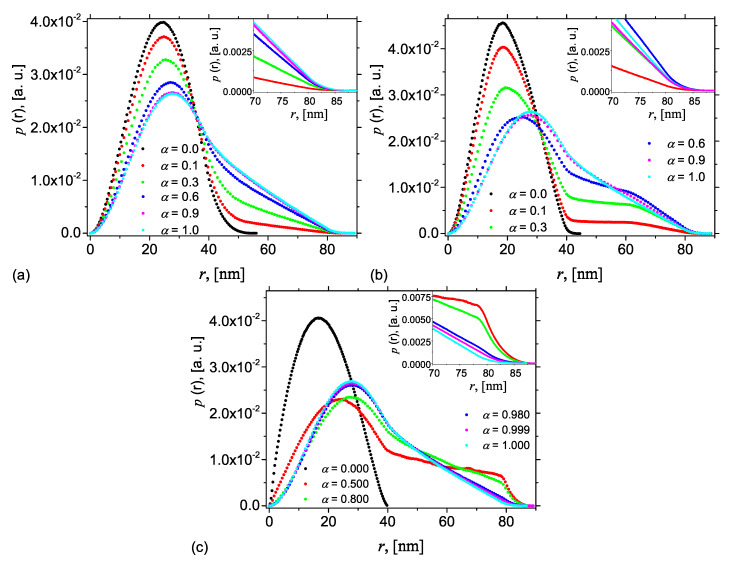
(Color online) Pddf p(r) from radially symmetric cylinders with diameter 2a=40 nm and height $2L_{0}=80$ nm calculated using the Monte-Carlo simulations, when Region 2 is found at various heights *h* above a middle-plane parallel to the bases: (**a**) h=0 nm; (**b**) h=20 nm; and (**c**) h=38 nm.

Figure 4b shows the pddfs when the height *h* is increased to 100 nm. This case reflects the situation when, although the two regions of AnJPs have different sizes, both can be considered as 3D objects. This behavior is similar to the previous case in Figure 4a with the difference that, at the inflection point $r≃L_{0}+h=240$ nm, the slopes of the pddfs increase. Therefore, the type of transition at this point is an indication of weather the AnJPs is symmetric of not. Figure 4c presents the case when h=137 nm, i.e., when $h≃L_{0}$. Thus, Region 2 is much smaller than the overall size of AnJP, which implies that h≪2a, and thus it can be assimilated to a 2D disk. This induces additional small irregular oscillations in the pddf when α<0.8, and with amplitudes slightly increasing with *r*. The inset in Figure 4c clearly shows these oscillations when α=0.5 and 0.8. Similar results are shown in Figure A1 for 2a=40 nm, $2L_{0}=80$ nm.

#### 3.2.1. The Scaling Approach

As shown above, features at different scales can be clearly seen in pddfs. To describe all these features also in the reciprocal space, in a unified way, here is presented a scaling description based on comparison of various power-law regimes to the various length scales of AnJPs. Thus, the power-law dependency of the scattering intensity on the scattering vector *q* is correctly predicted, as well as the crossover position (i.e., the transition point) between two such regimes. However, this approach does not describe the coefficients of these power-law decays and cannot account also for the oscillations present in the scattering curves. However, for practical purposes, these are not of concern since, in most cases, the investigated systems are not monodisperse.

Let us consider that the model-free scattering intensity of Region 1, i.e., of the cylinder with height *h* and diameter 2a, is of the following form:

(14) $$\langle |F_{1}\left(q\right){|}^{2}\rangle =\{\begin{array}{c} 1,q≲\frac{\pi }{L_{0}}\\ {\left(q\frac{L_{0}}{\pi }\right)}^{-2},\frac{\pi }{L_{0}}≲q≲\frac{\pi }{a}.\\ {\left(q\frac{\sqrt{aL_{0}}}{\pi }\right)}^{-4},\frac{\pi }{a}≲q \end{array}$$

**Figure 5:**
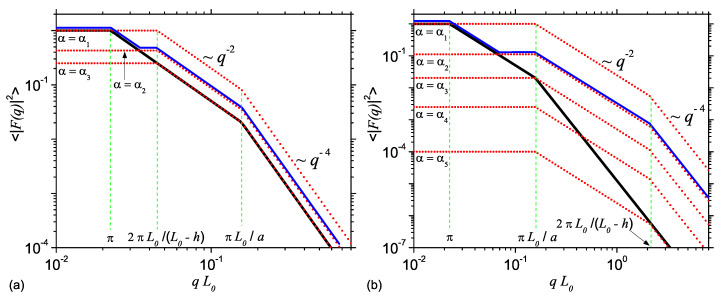
(Color online) Generic normalized SAS intensity from a cylinder, as a function of momentum transfer: (**a**) $L_{0}-h\geq 2a$. (**b**) $L_{0}-h<2a$. Black curve (continuous): SAS intensity $<|F_{1}{\left(q\right)|}^{2}>$ (Equation (14)) from a cylinder of length $2L_{0}$ and radius 2a. Red curves (discrete): SAS intensity $\left(1-\alpha \right)\alpha^{-1}<{\left|F_{2}\left(q\right)\right|}^{2}>$, at fixed values of contrast parameter α. The blue curves (slightly shifted vertically, for clarity) show the envelope of black and red curves corresponding to $F_{1}\left(\cdot \right)$ and $F_{2}\left(\cdot \right)$ at $\alpha =\alpha_{2}$.

Here, the coefficients of the power-law decays $q^{0}$, $q^{-2}$ and $q^{-4}$ are chosen such that they assure the continuity of the intensity at $q=\pi /L_{0}$ and π/a. Note that, according to the model shown in Figure 1, the relative values of $L_{0},a$ and *h* can be chosen in such a way that the AnJPs resemble either a 3D object (cylinder) or a 2D one (disk). The behavior of corresponding intensity is presented in Figure 5a (or Figure 5b)—black curves. The scattering intensity gives a succession of the type $q^{0}\to q^{-2}\to q^{-4}$ power-law decays. The transition points are at $\pi /L_{0}$ and π/a as expected (see Equation (14)).

A similar expression as in Equation (14) can also be written for Region 2. However, since, in this case, either the diameter (2a) or the height ($L_{0}-h$) can have relatively higher values, the intensities corresponding to the cases when $L_{0}-h>2a$ and $L_{0}-h<2a$ can be written as:

(15) $$\langle |F_{2}\left(q\right){|}^{2}\rangle =\{\begin{array}{c} 1,q≲\frac{2\pi }{L_{0}-h}\\ {\left(q\frac{L_{0}-h}{2\pi }\right)}^{-2},\frac{2\pi }{L_{0}-h}≲q≲\frac{\pi }{a},\\ {\left(q\frac{\sqrt{a\left(L_{0}-h\right)}}{\sqrt{2}\pi }\right)}^{-4},\frac{\pi }{a}≲q \end{array}$$

and

(16) $$\langle |F_{2}\left(q\right){|}^{2}\rangle =\{\begin{array}{c} 1,q≲\frac{2\pi }{a}\\ {\left(q\frac{a}{\pi }\right)}^{-2},\frac{\pi }{a}≲q≲\frac{2\pi }{L_{0}-h},\\ {\left(q\frac{\sqrt{a\left(L_{0}-h\right)}}{\sqrt{2}\pi }\right)}^{-4},\frac{2\pi }{L_{0}-h}≲q, \end{array}$$

respectively. These intensities are shown in Figure 5a,b (red dots), respectively, for various values of the contrast parameter α.

The common feature is the presence of a $q^{0}\to q^{-2}\to q^{-4}$ transition. When $L_{0}-h\geq 2a$, the beginning of the $q^{-2}$ decay is at $q=2\pi /(L_{0}-h)$ (<π/a). However, when $L_{0}-h<2a$, the beginning of $q^{-2}$ decay of Region 2 (at π/a) coincides with the end of the $q^{-2}$ decay of Region 1. This has important consequences on the overall intensity describing the AnJP. While in the former case, the upper limit is at $q=2\pi /(L_{0}-h)$, in the later case, this is extended up to $q=2\pi /(L_{0}-h)$. Thus, since the position of the transition points in Regions 1 and 2 does not coincide, for the contrast parameter values α ranging between $\alpha_{1}$ and $\alpha_{3}$ (shown in Figure 5a,b, respectively), the overall intensity of AnJP will be characterized by a more complex type of transition, of the form $q^{0}\to q^{-2}\to q^{0}\to q^{-2}\to q^{-4}$, as shown by the blue dash-dotted lines at $\alpha =\alpha_{2}$. The exact values of $\alpha_{1}$ and $\alpha_{3}$ depend on the geometrical parameters $2a,L_{0}$ and *h* of AnJP, and can be found from the conditions α=1/2 (with α given by Equation (5)), and by imposing the equality of the two terms containing $F_{1}\left(\cdot \right)$ and $F_{2}\left(\cdot \right)$ occurring in Equation (6) at the point q=π/a, respectively.

### 3.3. Small-Angle Scattering Intensity

By using Equation (9), the form factor of Region 2 can be written as:

(17) $$F_{2}\left(q,\psi \right)=\frac{1}{V_{2}}\int_{h}^{L_{0}}e^{iq\mu z}dz\int_{0}^{a}rdr\int_{0}^{2\pi }e^{iqr\cos\theta \sqrt{1-\mu^{2}}}d\theta ,$$

where μ=cosψ, ψ is the angle between positive direction of axis and scattering vector q.

Therefore, after a little algebra, one finds that the form factor of the AnJPs can be written as:

(18) $$\begin{array}{cc} F\left(q,\psi \right) & =\alpha \frac{\sin\left(q\mu L_{0}\right)}{q\mu L_{0}}\frac{2J_{1}\left(qa\sqrt{1-\mu^{2}}\right)}{qa\sqrt{1-\mu^{2}}}\\ \; & +a\beta q^{-1}J_{1}\left(qa\sqrt{1-\mu^{2}}\right)\int_{h}^{L_{0}}\cos\left(q\mu z\right)dz\\ \; & +ia\beta q^{-1}J_{1}\left(qa\sqrt{1-\mu^{2}}\right)\int_{h}^{L_{0}}\sin\left(q\mu z\right)dz, \end{array}$$

where the first term corresponds to the form factor of Region 1 (i.e., cylinder of height $2L_{0}$ and radius 2a), $\beta =2\pi \left(1-\alpha \right){\left(V_{2}\right)}^{-1}{\left(1-\mu^{2}\right)}^{-1/2}$ and $J_{1}\left(\cdot \right)$ is the first order Bessel function of the first kind. Note that $F\left(\cdot \right)$ depends only on the angle ψ, and thus the function $f\left(\cdot \right)$ in Equation (3) is independent of the azimuthal angle ϕ. Therefore, the intensity from AnJPs is calculated according to:

(19) $$\frac{I\left(q\right)}{I\left(0\right)}=\frac{1}{2}\int_{0}^{\pi }{\left|F\left(q,\psi \right)\right|}^{2}\sin\psi d\psi .$$

**Figure 6:**
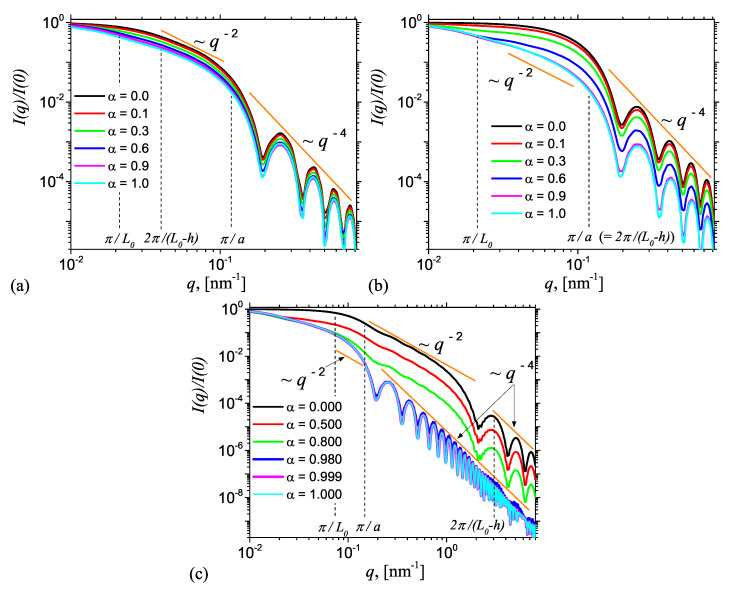
(Color online) SAS intensity from radially symmetric JC with diameter 2a=40 nm and height $2L_{0}=280$ nm calculated using Equation (19), when Region 2 is found at various heights *h* above a middle-plane parallel to the bases: (**a**) h=0 nm; (**b**) h=100 nm; and (**c**) h=137 nm. α is the contrast parameter given by Equation (5).

Figure 6 shows the corresponding intensities from AnJPs as a function of height *h* and of the contrast parameter α given by Equation (5). Their numerical values were chosen such that all the cases discussed in Figure 5 are addressed. The common feature is the presence of a Guinier regime, i.e., $I\left(q\right)\propto q^{0}$ for $q≲\pi /L_{0}$, an intermediate regime where $I\left(q\right)\propto q^{-2}$ for $\pi /L_{0}≲q≲\pi /a$ when $L_{0}-2h\geq 2a$ and $\pi /L_{0}≲q≲2\pi /\left(L_{0}-h\right)$ when $L_{0}-h<2a$, followed by Porod regime where $I\left(q\right)\propto q^{-4}$. All the curves in Figure 6 have been reproduced also by performing the Fourier transform given by Equation (10) of the pddf obtained from Monte-Carlo simulations shown in Figure 4. The results are presented in the Appendix A (Figure A3) and show a very good agreement between the analytic curves and numerical simulations. This confirms the validity of the developed models.

Figure 6a presents the results when h=0, corresponding to the case when AnJPs resemble cylinders in which the two regions of different SLDs are situated symmetrically with respect to the middle plane parallel to the bases. For this case, the differences between scattering curves when α varies from zero to one, i.e., when the structure of AnJPs changes from a cylinder of height $L_{0}$ and radius 2a to a cylinder of height $2L_{0}$ and the same radius, are not very pronounced. The exception is the end of the Guinier regime, which becomes smaller with increasing α due to the increase of the contribution of Region 1. As a consequence, the length of $q^{-2}$ decay increases with contrast parameter α, and thus it is more reliable for an experimental determination of the cylinder size. Note that the minima positions in the Porod regime are the same, indicating that, in the whole range of α values, the AnJPs preserve their cylindrical structure.

Figure 6b treats the case when the two sides of AnJP are now different in size, but still both resemble 3D objects. The numerical value used for the height *h* is 100 nm, and the values of the contrast parameter α are the same as in Figure 6a. Generally, the results show a transition of the type $q^{0}\to q^{-2}\to q^{-4}$. However, the differences between the scattering curves at fixed values of α, are more pronounced in both the Guinier and Porod regimes, due to differences in the overall dimensions of the two regions. As α→0, the dominant contribution is given by Region 2, i.e., the cylinder of height $L_{0}-h=40$ nm and diameter 2a=40 nm, while for α→1, the dominant contribution comes from Region 1 (see Equation (6)), i.e., the cylinder of height $2L_{0}=280$ nm, and which has the same diameter as Region 2. Therefore, in the former case, we have an almost completely globular particle, and thus the $q^{-2}$ decay is hardly visible (see black curve). However, as α is increased from 0 to 1, the $q^{-2}$ decay becomes more clearly visible, with its maximum length occurring at α=1. In addition, the minima positions in Porod regime coincide for all values of α.

Figure 6c presents the results for a third important case, when the height h=137 nm is almost equal to $L_{0}$ (=140 nm). Therefore, Region 2 becomes a disk of height $L_{0}-h=8$ nm and diameter 40 nm, which closely resemble a 2D structure. Thus, one expects that, for some particular value of α, significant changes in the behavior of the SAS curves shall occur, reflecting the transition from 3D to 2D-like structures. Indeed, numerical investigations show that for the chosen parameters ($L_{0},h$ and *a*) a transition occurs at 0.8≲α≲0.98. When α≲0.8 the scattering curves show a long $q^{-2}$ power-law decay, i.e., for $\pi /L_{0}≲q≲2\pi /\left(L_{0}-h\right)$, followed by a Porod regime, thus reflecting the dominant contribution of the 2D-disk, while at α≳0.98 the length of the $q^{-2}$ decay is significantly reduced, and replaced by a Porod regime, as an effect of the dominance of Region 1, i.e., the 3D cylinder (of height $2L_{0}$ and diameter 2a). Thus, the interplay between the relative sizes of the two regions of AnJPs, and the strength of their SLDs on the scattering curve, can be controlled through the values of the scattering parameter α. Note that, in Figure 6b,c, the scattering curves in the Porod regime are significantly shifted up with decreasing the values of α, and this can be used to extract information about the specific surface [29].

### 3.4. Radius of Gyration

One of the most important structural parameters which can be obtained from SAS data shown in Figure 4 is the radius of gyration $R_{g}$ of AnJP. This is a measure of its overall size, and is related to the average of square center-of-mass distances inside the particle, weighted by the SLD. Here, $R_{g}$ is obtained from Equation (11), which makes use of the whole available data in the scattering intensity. The advantage over other methods such as the Guinier plot, is that in the former case is used all the available experimental data.

**Figure 7:**
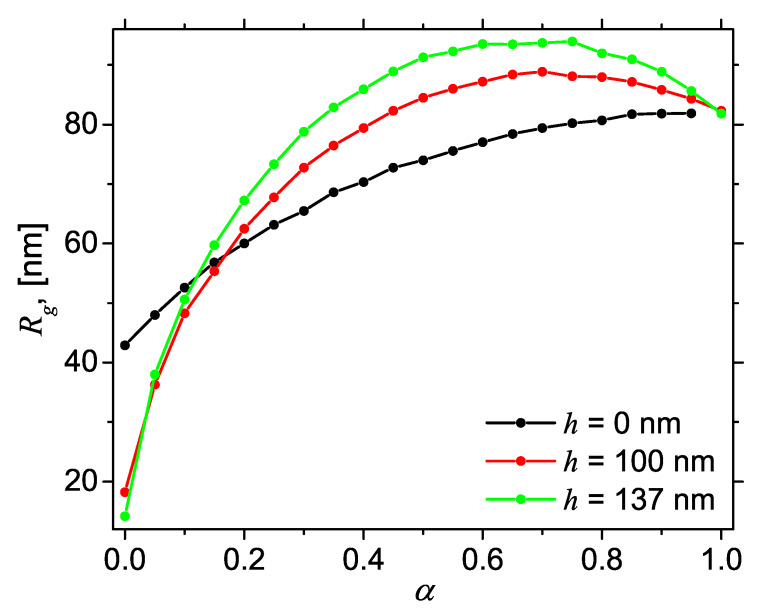
(Color online) Radius of gyration $R_{g}$ (black) of AnJPs with diameter 2a=40 nm and height $2L_{0}=280$ nm as a function of dimensionless parameter α (Equation (5)) for fixed values of the height *h* above middle-plane parallel to the bases (see Figure 3 for details): (Black) h=0 nm; (Red) h=20 nm; and (Green) h=137 nm.

**Figure A2:**
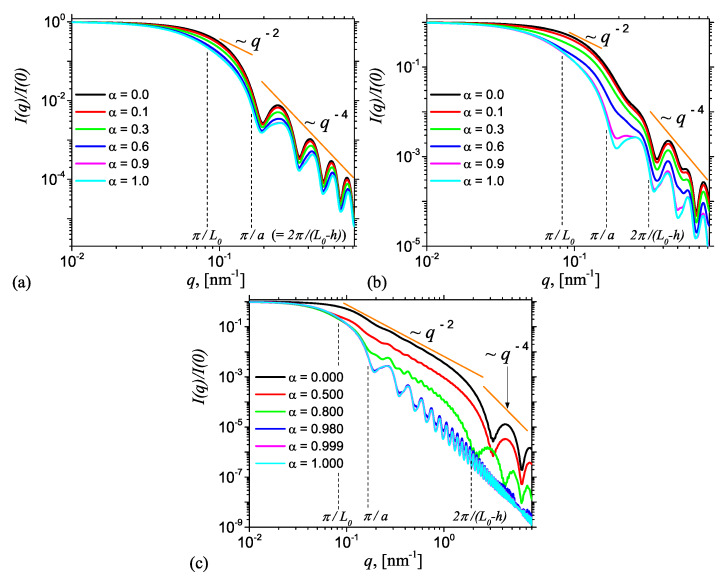
(Color online) SAS intensity from radially symmetric cylinders with diameter 2a=40 nm and height $2L_{0}=80$ nm, when Region 2 is found at various heights *h* above a middle-plane parallel to the bases: (**a**) h=0 nm; (**b**) h=20 nm; and (**c**) h=38 nm.

**Figure A3:**
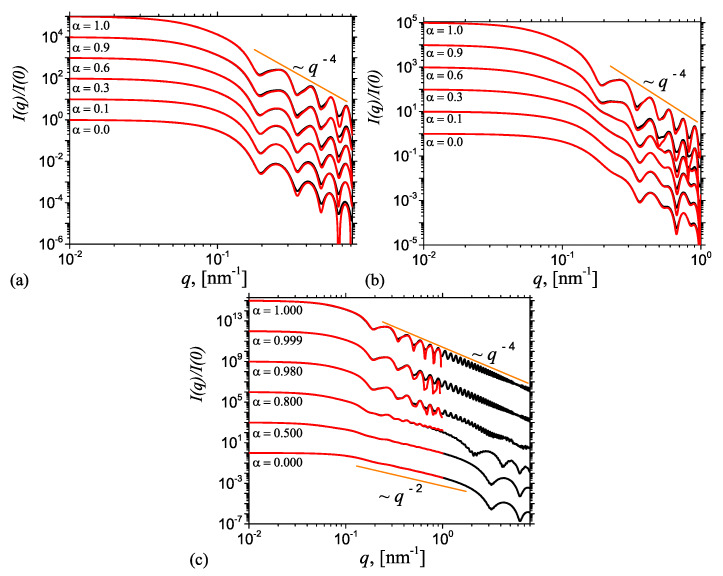
(Color online) Comparison between theoretical curves (black; Equation (19)) and Monte-Carlo simulations (red; Equation (10)) for AnJPs with height $2L_{0}=80$ nm and diameter 2a=40 nm, at: (**a**) h=0 nm; (**b**) h=20 nm; and (**c**) h=38 nm, for different values of the contrast parameter α. For clarity, the curves corresponding to α≠0 are shifted vertically by a factor of $10^{i}$ (**a**,**b**) and $10^{3i}$ (**c**), with i=1,⋯,5, in increasing order of the values of α.

Figure 7 shows the variation of $R_{g}$ with the contrast parameter α at the same values of height *h*, as in Figure 4 and Figure 6. One can distinguish two main types of behavior: when h=0, $R_{g}$ increases asymptotically to a maximum value corresponding to α=1 (black curve), while, for h≠0, the variation of $R_{g}$ with α has a downward parabola-like behavior (red and green curves). However, at α=1, all the curves have a common value, irrespective of the height *h*. This property arises since, regardless of the value of *h*, when α=1, we always have a cylinder of diameter 2a and height $2L_{0}$. By using the values 2a=40 nm and $2L_{0}=140$ nm in Equation (12), one obtains $R_{g}≃82$ nm, which is in very good agreement with the numerical value of $R_{g}$ given in Figure 7. This confirms the validity of the proposed approach based on Monte Carlo simulations, to obtain the radius of gyration of AnJPs. Similar results are shown in Figure A2 for 2a=40 nm, $2L_{0}=80$ nm.

The interplay between geometrical and chemical asymmetries of AnJPs when h≠0 is manifested through a decrease of $R_{g}$ at α=0 and an increase of the parabola maximum with *h*. Thus, if one measures $R_{g}$ vs. α for various values of the height *h*, then one can determine the relative degree of geometrical asymmetry of AnJPs, that is the higher is the value of $R_{g}$ the more different is the structure compared with a AnJP in which h=0. Similarly, a plot of $R_{g}$ vs. *h* at various values of α can be used to determine the relative degree of chemical asymmetry.

### 3.5. Contrast Variation

As shown in Equation (5), besides the volumes $V_{2}$ and $V_{1}$ of Regions 2 and Region 1, respectively, the contrast parameter α depends also on the SLD $\rho_{2}$ and $\rho_{1}$ of these regions, as well as on the SLD $\rho_{0}$ of the solvent/matrix in which the AnJPs are embedded. Since this parameter controls the relative contributions of these regions to the total scattering intensity (see Equation (6)), it leads to a succession of power-law decays with various scattering exponents, as discussed in Section 3.2.1 and schematically depicted in Figure 5.

When we are interested in determining the shape, size or the relative arrangement of the different regions composing the AnJPs, then a contrast variation needs to be performed. Generally, for Janus particles consisting from complex, possibly self-similar (fractal) regions, the contrast variation can be applied beyond the Guinier regime. This would also allow distinguishing among various types of structural organization in AnJPs, such as if one region is embedded (immersed) into another one, or if they are non-overlapping [30]. However, for the AnJPs discussed here, both regions are Euclidean objects, and a contrast variation at q=0 can be performed.

Experimentally, when neutrons are used, the contrast variation can be achieved by preparing a number of several samples which differ in the SLD of each region, as well as in the SLD of the solvent/matrix. Then, the variation of each of these three parameters ($\rho_{0},\rho_{1}$, and $\rho_{2}$) has its own imprint on the behavior of scattering intensity at q=0, through Equation (7). Therefore, in the following, we analyze each of these contributions individually, that is:

- Case I: $\rho_{1}$ and $\rho_{2}$ are fixed, and $\rho_{0}$ is variable.
- Case II: $\rho_{0}$ and $\rho_{2}$ are fixed, and $\rho_{1}$ is variable.
- Case III: $\rho_{0}$ and $\rho_{1}$ are fixed, and $\rho_{2}$ is variable.

Thus, by using the explicit values of $V_{2}$ given by Equation (13), and $V_{1}=2\pi L_{0}a^{2}$ in Equation (5), Equation (7) can be rewritten as:

(20) $$\frac{I(0)}{nV_{1}^{2}}=\frac{{\left(L_{0}\left(\rho_{0}-\rho_{2}\right)+h\left(\rho_{0}-2\rho_{1}+\rho_{2}\right)\right)}^{2}}{{\left(h+L_{0}\right)}^{2}}.$$

Then, the position of the contrast matching point for Case I can be found by taking the derivative with respect to $\rho_{0}$ of Equation (20), i.e.,

(21) $$\frac{1}{nV_{1}^{2}}\frac{\partial I(0)}{\partial \rho_{0}}=\frac{2\left(L_{0}\left(\rho_{0}-\rho_{2}\right)+h\left(\rho_{0}-2\rho_{1}+\rho_{2}\right)\right)}{h+L_{0}},$$

and then equating the last expression with zero. This operation gives the position of the contrast matching point $\rho_{0}$, as:

(22) $$\rho_{0}=\frac{2h\rho_{1}-h\rho_{2}+L_{0}\rho_{2}}{h+L_{0}}.$$

Similarly, for Case II, one can find the derivative with respect to $\rho_{1}$, i.e.,

(23) $$\frac{1}{nV_{1}^{2}}\frac{\partial I(0)}{\partial \rho_{1}}=-\frac{4h\left(L_{0}\left(\rho_{0}-\rho_{2}\right)+h\left(\rho_{0}-2\rho_{1}+\rho_{2}\right)\right)}{{\left(h+L_{0}\right)}^{2}},$$

and the position of the contrast matching point $\rho_{1}$ is:

(24) $$\rho_{1}=\frac{h\rho_{0}-L_{0}\rho_{0}+h\rho_{2}+L_{0}\rho_{2}}{2h}.$$

For Case III, the derivative with respect to $\rho_{2}$ is:

(25) $$\frac{1}{nV_{1}^{2}}\frac{\partial I(0)}{\partial \rho_{2}}=\frac{2\left(h-L_{0}\right)\left(L_{0}\left(\rho_{0}-\rho_{2}\right)+h\left(\rho_{0}-2\rho_{1}+\rho_{2}\right)\right)}{{\left(h+L_{0}\right)}^{2}},$$

and the position of the contrast matching point $\rho_{2}$ is:

(26) $$\rho_{2}=\frac{-h\rho_{0}-L_{0}\rho_{0}+2h\rho_{1}}{h-L_{0}}.$$

**Figure 8:**
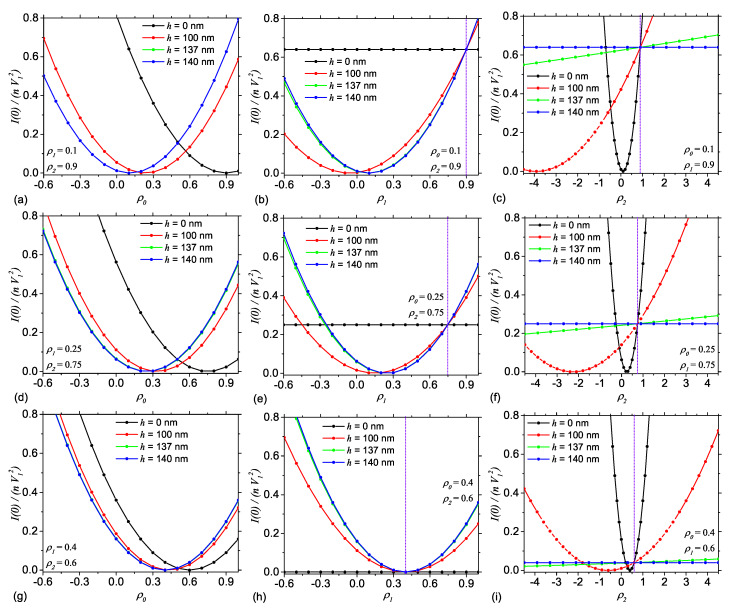
(Color online) Scattering intensities from AnJPs at q=0, as a function of SLD of different regions, for fixed values of height *h*: (**a**,**d**,**g**) variation with solvent density $\rho_{0}$; (**b**,**e**,**h**) variation with solvent density $\rho_{1}$; and (**c**,**f**,**i**) variation with solvent density $\rho_{2}$.

Figure 8a,d,g shows the contrast variation for Case I, with $\rho_{1}=0.1$ and $\rho_{2}=0.9$, $\rho_{1}=0.25$ and $\rho_{2}=0.75$, and $\rho_{1}=0.4$ and $\rho_{2}=0.6$, respectively, for various values of the height *h*. In all cases, the variation of I(0) with $\rho_{0}$ have a parabola-like behavior, with minima given by Equation (22). The common feature is that for each values of the pair $\left(\rho_{1},\rho_{2}\right)$, the minima $\rho_{0}^{\min}$ satisfy the condition $\rho_{1}<\rho_{0}^{\min}<\rho_{2}$. These lower and upper limits correspond to $h=L_{0}$ and h=0, respectively. As the value between $\rho_{1}$ and $\rho_{2}$ decreases, the differences between the positions of the contrast matching points also decrease. In the limiting case when $\rho_{1}=\rho_{2}$, we have an AnJP with a single region, and thus the variation of I(0) is independent of the height *h*, as expected.

Figure 8b,e,h shows the contrast variation for Case II, with $\rho_{1}=0.1$ and $\rho_{2}=0.9$, $\rho_{1}=0.25$ and $\rho_{2}=0.75$, and $\rho_{1}=0.4$ and $\rho_{2}=0.6$, respectively. Note that, at h=0, the parabola-like behavior from the previous case is replaced by a straight line (black curve). This is clear from Equation (24), which has no solution when h=0. The line intersects the I(0)-axis at ${\left(\rho_{0}-\rho_{2}\right)}^{2}$, as indicated by Equation (20). However, when h≠0 the variation of I(0) with $\rho_{1}$ has a parabola-like behavior, with minima $\rho_{1}^{\min}$ given by Equation (24). For a given pair of values $\left(\rho_{0},\rho_{2}\right)$, we have $\rho_{1}^{\min}<\rho_{0}$ at $h=L_{0}$. By decreasing the values of *h* the minima are shifted to the left, and the parabola opens up, leading to the straight line (black), in the limit h=0, as discussed above. In addition, when $\rho_{0}\to \rho_{2}$, all the parabolas reduce to a single one with minimum at $\rho_{1}^{\min}=\rho_{0}$, to which is tangent the straight line corresponding to h=0 (see an approximation in Figure 8h). The position of the common point is indicated by the vertical violet-dotted line. Such a common point exists for any pair of $\left(\rho_{0},\rho_{2}\right)$ values, and the higher the difference between $\rho_{0}$ and $\rho_{2}$, the point is more shifted to the right (see also vertical lines in Figure 8b,e). This common points arise when $\rho_{1}=\rho_{2}$, that is when AnJP consists from a single region.

Figure 8c,f,i shows the contrast variation for Case III, with $\rho_{0}=0.1$ and $\rho_{1}=0.9$, $\rho_{0}=0.25$ and $\rho_{1}=0.75$, and $\rho_{0}=0.4$ and $\rho_{1}=0.6$, respectively. The overall behavior is similar to the one in Case II, when generally we have a set of parabolas (red, green, and blue curves), while for a limiting value of *h*, here at $h=L_{0}$, we have a straight line (black curve). The minima of the parabolas are given by Equation (26) and satisfy the condition $\rho_{2}^{\min}<\rho_{1}$. In the limiting case when $\rho_{2}^{\min}=\rho_{0}$, the solvent matches Region 2, and thus at h=0 the position of the contrast matching point is right-most, for a given pair $\left(\rho_{0},\rho_{1}\right)$. By increasing the value of *h*, the position of minima are shifted to the left. Note also the presence of a common point to all curves, for a given pair of values $\left(\rho_{0},\rho_{1}\right)$, where the forward scattering intensity is $I\left(0\right)={\left(\rho_{0}-\rho_{1}\right)}^{2}$. Their position on $\rho_{2}$-axis are indicated by vertical violet dotted lines. As for Case II, these points correspond to AnJPs consisting from a single region.

The above observations illustrate that, in all three cases, contrast variation may be used to obtain the contrast matching points. The parabola describing the overall behavior of I(0) can be further used as basic functions in a more detailed analysis, together with the values of the radii of gyration (see Figure 7) for a more detailed analysis, to extract additional information such as the relative arrangement of higher and lower density regions within AnJP, with respect to their center of mass, or the deviation of the center of masses of each region of AnJP from the center of mass of the whole AnJP.

## 4. Conclusions

In this work is suggested a method for a detailed structural characterization of AnJPs with tunable geometrical and chemical asymmetries. The method involves calculation of the scattering intensity and performing a contrast variation, which is then followed by extraction of the relevant structural parameters.

The model of AnJP studied here is based on a cylinder composed from two regions with different SLDs and heights, arranged on top of each other along the cylinder axis. This is a generalization of the semi-cylindrical model in which two cylinders of different SLD but the same height are arranged on top of each other.

The obtained scattering intensity shows a complex behavior which involves a succession of power-law decays of the type $q^{0}\to q^{-2}\to q^{-4}$. To explain this behavior, a simple scaling approach is used which takes into account the contribution of each individual region of AnJP to the total scattering intensity. It is shown that this allows a detailed characterization of each individual region, including shape, size, and the specific surface. This involves a combined analysis of the slope and the limits of the power-law decays in which a particular value of the slope holds constant.

The contrast variation is applied to each individual region as well as for the solvent/matrix. Analytic expressions for contrast matching points are derived for each case. The information obtained from the contrast variation can be used to extract additional structural properties such as the relative arrangement of each region inside AnJP or the deviations of the distances between the center of masses of each region from the position of the center of mass of the whole AnJP.

The effectiveness of the proposed method shall be clear also for more complex systems, especially for those consisting from complicated regions and for which calculating analytic expression of the form factor can be a tedious task, for multiphase systems in which one or more regions is/are completely immersed into another region of different SLD, etc. Thus, the correlations between structural and physical/chemical properties of complex multiphase nano/micro scales systems can be assessed under a wide range of geometrical structures with various chemical compositions. 

## Appendix A. Monte Carlo Simulations of SAS Intensity

Pddfs and scattering intensities (theoretical and from Monte Carlo simulations) for different structural parameters.
